# Contribution of NKX2-3 Polymorphisms to Inflammatory Bowel Diseases: A Meta-Analysis of 35358 subjects

**DOI:** 10.1038/srep03924

**Published:** 2014-01-29

**Authors:** XiaoCheng Lu, Linjun Tang, Kai Li, JinYu Zheng, Penglai Zhao, Yi Tao, Li-Xin Li

**Affiliations:** 1 Department of Neurosurgery, The First Affiliated Hospital of Nanjing Medical University, 300 Guangzhou Road, Nanjing, Jiangsu, 210029, China; 2 These authors contributed equally to this work.

## Abstract

Polymorphisms in NKX2-3 gene have been inconsistently associated with Crohn's disease (CD) and ulcerative colitis (UC). To generate large-scale evidence on whether NKX2-3 polymorphisms are associated with CD or UC susceptibility we have conducted a meta-analysis of 17 studies involving 17329 patients and 18029 controls. A significantly increased CD or UC risk was observed in persons carrying a G allele at rs10883365 polymorphism (A/G) compared with those with a A allele. (OR = 1.226, 95%CI: 1.177–1.277 and OR = 1.274, 95%CI: 1.175–1.382 respectively). In the subgroup analysis, a significantly increased CD risk was found in both Europeans and Asians. For rs11190140 polymorphism (C/T) and CD risk, the risk estimate for the allele contrast was OR = 1.201 (1.136–1.269). This meta-analysis provided a robust result that persons with a G or T allele may have a moderately increased risk of CD, and suggested that rs10883365 polymorphism was also a candidate gene polymorphism for UC susceptibility.

Inflammatory bowel diseases (IBDs) are chronic inflammatory disorders characterized by chronic relapsing inflammation of the gastrointestinal tract that affect 0.1% of Western populations, comprising two major forms, Crohn's disease (CD) and ulcerative colitis (UC)^1^. In Crohn's disease the inflammation is often transmural, whereas in ulcerative colitis the inflammation is typically confined to the mucosa. Additionally, Crohn's disease can be associated with intestinal granulomas, strictures, and fistulas, but these are not typical findings in ulcerative colitis. Although our understanding of disease pathogenesis remains incomplete, accumulating evidence suggests that that IBD is a complex, multifactorial disease partly determined by a genetic predisposition^2^. Strong familial aggregation, twin studies, and established genetic associations^3^,^4^,^5^. indicate that there is a genetic component to the disease susceptibility in IBD. Recently, sequence variations associated with IBD have been reported for several genes, including NOD2, IL23R, IRGM, ATG16L1, PTPN2, and NK2 transcription factor related and locus 3 (NKX2-3)^6^,^7^,^8^,^9^,^10^,^11^.

NKX2-3, located on 10q24, is a member of a family of genes that encodes transcription factors containing homeodomains and, therefore, is implicated in basic developmental functions. During development, NKX2-3 is expressed in midgut and hindgut mesoderm and spleen, as well as in pharyngeal endoderm^12^,^13^. The association between the NKX2-3 polymorphism and susceptibility of IBD was first reported in Caucasian patients^14^. After the first report of the association, several studies confirmed the association of tag-SNPs (rs10883365 and rs1190140) in the NKX2-3 gene with CD^15^,^16^ as well as with UC in Caucasian or Asian populations^17^,^18^,^19^. However, several studies could not replicate the genetic association between IBD and NKX2-3 polymorphsims^15^,^20^,^21^.

Thus, a quantitative synthesis may help to provide clearer evidence on the association of such genetic polymorphisms with IBD. In the present study, we conducted a meta-analysis of all eligible studies to quantitatively assess the associations between three common polymorphisms (rs10883365 and rs11190140) in the NKX2-3 gene and IBD susceptibility.

## Results

### Characteristics of the included studies

**Figure 1:**
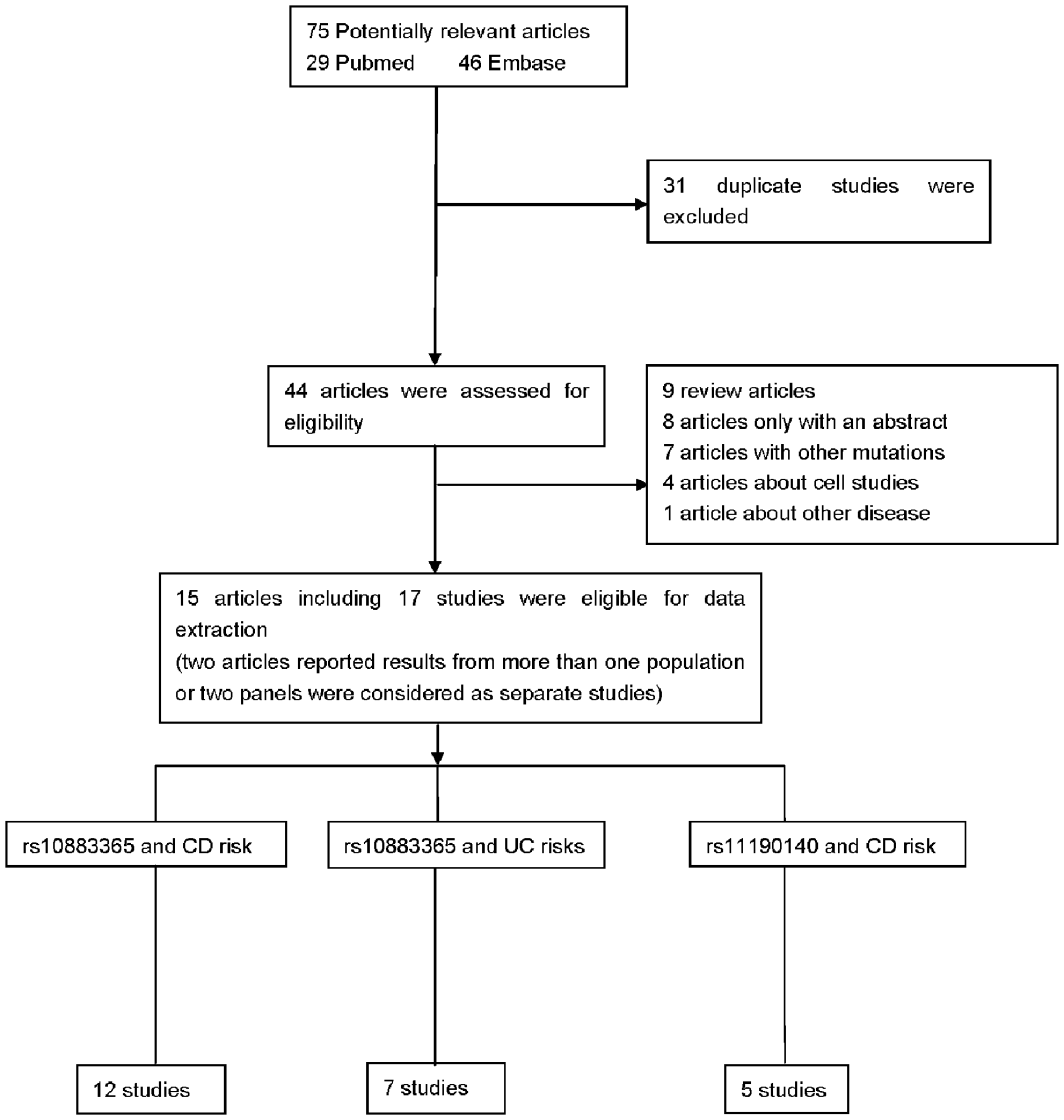
Study selection procedures for a meta-analysis of NKX2-3 polymorphisms and risk of CD or UC. NKX2-3: NK2 transcription factor related and locus 3; CD: Crohn's disease; UC: ulcerative colitis.

The combined search yielded 75 references, of which 31 were duplicate studies, 9 were reviews, 4 were about cell studies, 8 were only with abstracts, 7 reported other mutations, 1 reported other disease. Finally, a total of 15 articles were finally included. Among them, one publication^15^ contained data on two different subpopulations, one^16^ included Wellcome Trust Case Control Consortium (WTCCC) samples and replication Crohn's disease (RCD) samples, and we treated them independently. In total, 17 studies comprising 17329 cases and 18029 controls were included in the present meta-analysis^11^,^15^,^16^,^17^,^18^,^19^,^20^,^21^,^22^,^23^,^24^,^25^,^26^,^27^,^28^. The 17 separate studies consisted of 13 European and 4 Asian. The distribution of genotypes in the control groups of all studies was in agreement with HWE except for 1 study^19^. Summaries of all included studies were summarized in Table 1, and the flow chart of study selection process was shown in Figure 1.

### Quantitative synthesis

#### Crohn's disease

**Figure 2:**
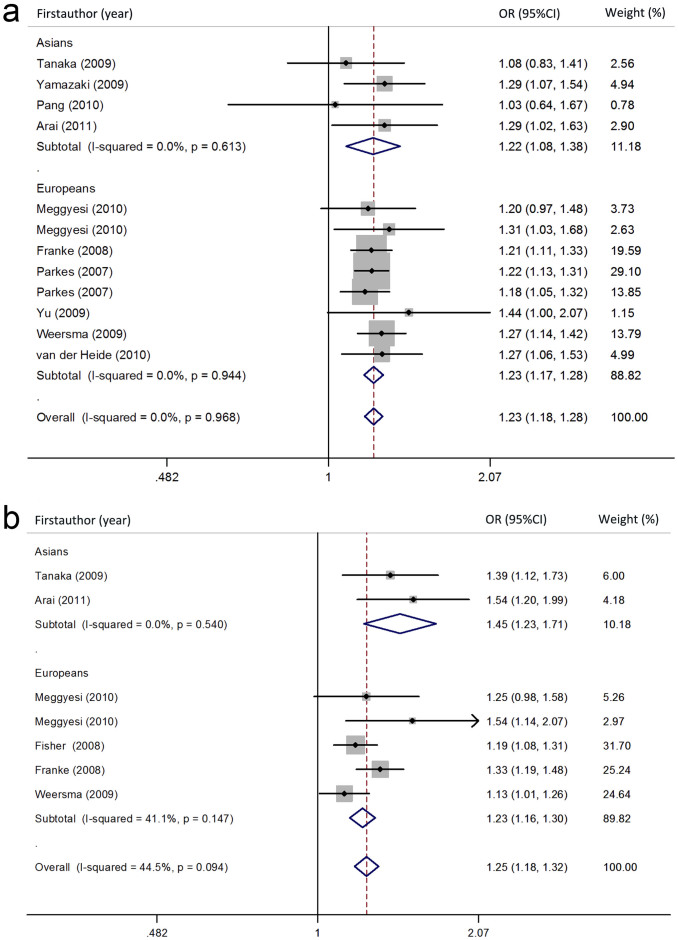
OR estimates with the corresponding 95% CI for the association between rs10883365 polymorphism in NKX2-3 gene and CD or UC risk. (a): rs10883365 polymorphism and CD risk (G vs. A), (b): rs10883365 polymorphism and UC risk (G vs. A). The sizes of the squares reflect the weighting of included studies. OR: odds ratio; CI: confidence interval.

The summary of meta-analysis for the NKX2-3 polymorphisms with CD is shown in Table 2, Figure 2A and Supplementary Figure S1. Regarding rs10883365 polymorphism, the results of combined analyses comprising 8699 cases and 13540 controls revealed a significantly increased risk of CD in all genetic models. In addition, the OR was 1.481 (1.351–1.623) in carriers of two risk G alleles compared with non-risk allele carriers (GG vs AA), which was higher than the risk of one G allele carriers (GA vs AA, OR = 1.141 (1.055–1.234), suggesting a dose–response with increasing number of the variant allele. In the subgroup analysis, significantly increased risks were found both among European and Asian population. No between-study significant heterogeneity was observed in all genetic models.

A total of 5484 patients and 4863 controls were investigated for rs11190140 variant, a significant association was found in all genetic models. (see Supplementary Fig. S1) Similar to rs10883365, the OR (OR = 1.485, P < 0.001) in carriers of 2 risk alleles was higher than that (OR = 1.155, P < 0.001) in those of 1 risk allele. No between-study heterogeneity was detected in any genetic models of rs11190140 variant and CD risk.

#### Ulcerative colitis

Seven studies with 4996 UC patients and 5479 controls for rs10883365 polymorphism were investigated. Meta-analysis findings of associations between rs10883365 in NKX2-3 gene and the risk of UC were shown in Table 3 and Figure 2B. Significantly increased UC risk was observed in all comparisons (G vs A: OR = 1.274 (1.175–1.382), GG vs AA: OR = 1.672 (1.474–1.896), GA vs AA = 1.207 (1.084–1.343), dominant model: OR = 1.342 (1.213–1.485), and recessive model: OR = 1.470 (1.325–1.630)). (Fig. 2B) When stratified by ethnicity, significant association was found both in European and Asian subgroups except for one genetic model in Asian (GA vs AA: OR = 1.260 (0.971–1.634)). No heterogeneity was detected in major genetic models.

### Sensitivity analyses and cumulative meta-analysis

**Figure 3:**
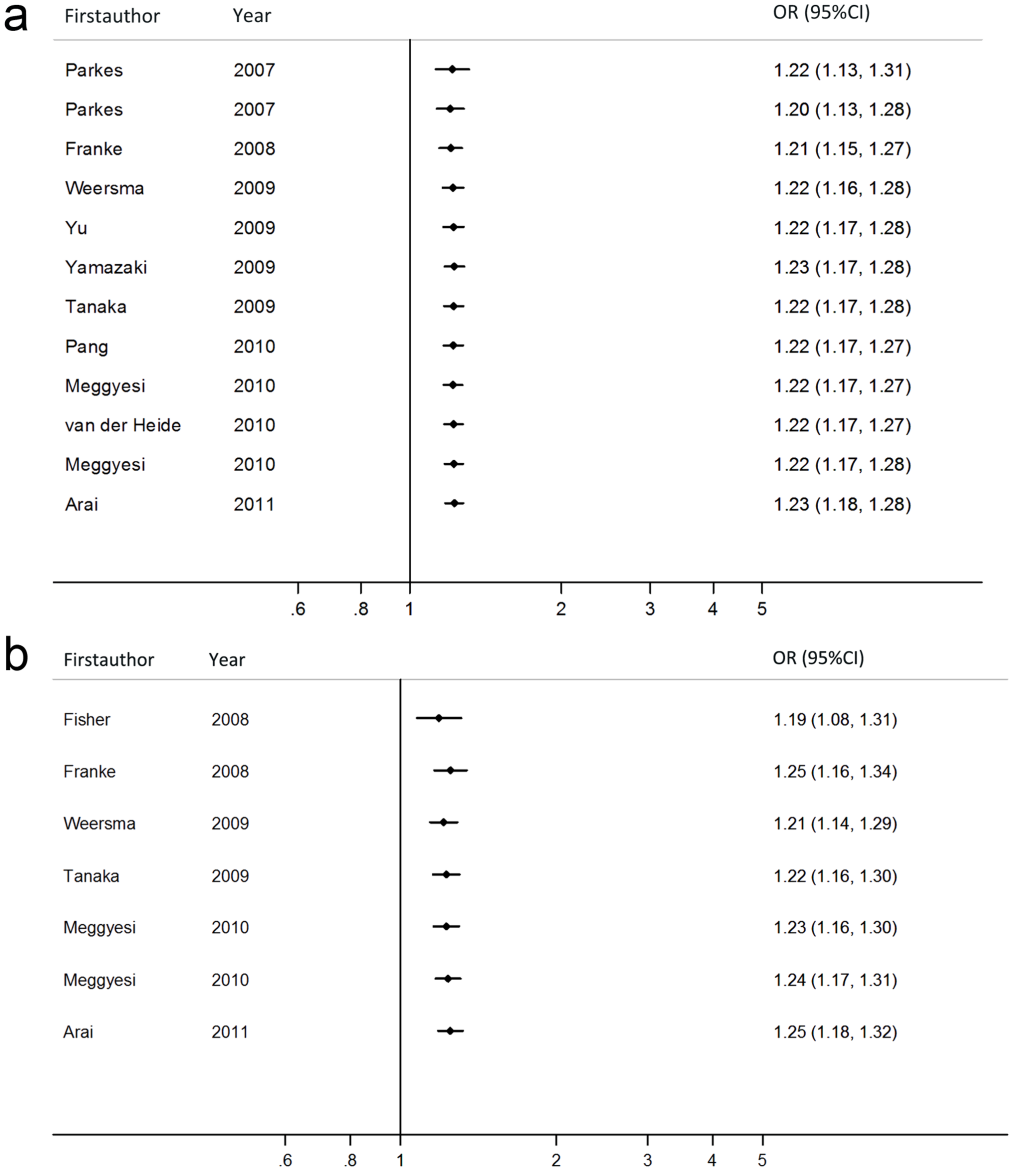
Cumulative meta-analysis on the association between rs10883365 polymorphism and CD or UC risk. (a): rs10883365 variant and CD risk (G vs. A); (b): rs10883365 variant and UC risk (G vs. A). Pooled OR estimates with the 95% CI as information accumulates at the end of each year (left column). CD: Crohn's disease; UC: ulcerative colitis; OR: odds ratio; CI: confidence interval.

Sensitivity analysis showed no single study qualitatively changed the pooled ORs. (see Supplementary Fig. S2 and S3) Moreover, there was a study which deviated from HWE, when excluded, the estimated pooled OR still did not change at all, indicating that the results of this meta-analysis were high stable. (Table 2 and 3) In the cumulative meta-analysis, the pooled ORs tended to be stable and the associations tended toward significant associations with accumulation of more data over time between rs10883365 or rs11190140 variant and CD risk, as well as between rs10883365 and UC risk (see Fig. 3 and Supplementary Fig. S4).

### Publication bias

Funnel plots and Egger's test were performed to assess publication bias. No publication bias was detected for rs10883365 polymorphism. (G vs A: t = −0.04, p = 0.966 and t = 1.56, p = 0.181 in CD and UC, respectively). Similarly, no publication bias was detected for T vs C contrast of rs11190140 polymorphism in CD. (see Supplementary Fig. S6) As shown in Supplementary Figure S5, the shapes of the funnel plots did not indicate any evidence of obvious asymmetry for rs10883365 variant and CD or UC risk.

## Discussion

Presently the mechanisms of the etiology and progression of IBD are far from clear. Several genes have been identified to be associated with IBD risk, including NOD2, NKX2-3 and IL-23. Recently, genome-wide association studies (GWAS) have identified SNPs implicating hundreds of replicated loci for common traits and becomes a powerful tool to detect the susceptibility genes in the IBD diseases^16^,^29^,^30^. Several GWAS and GWAS meta-analysis have provided strong evidences for the association between NKX2-3 single nucleotide polymorphisms (SNPs) (rs6584283 and rs4409764) and risk of IBDs^31^,^32^,^33^. However, two common variants (rs10883365 and rs11190140) in NKX2-3 gene were not included in the GWAS meta-analyses.

A meta-analysis can combine results from individual studies to overcome the limitation of small sample sizes and inadequate statistical power, and produce a single estimate of the major effect. Recently, accumulated meta-analysis has been performed to investigate the association of genetic variants with susceptibility to CD or UC. Polymorphisms in several genes, including ATG16L1 T300A^34^, TGF-α G308A^35^, MIF G173C^36^, OCTN1 C1672T^37^, CD14 C260T^38^ and MDR1 C3435T^39^, were identified as risk factors of CD or UC. Patients with mutant allele of NOD1 rs6958571^40^ and 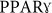 Pro12Ala^41^ might have a decreased susceptibility to IBD. Additionally, some genetic variants were not association with CD or UC risk, such as MDR1 C1236T^39^, IL-10 G1082A^42^, and IL-18 A607C^43^. Therefore, we saw the need to perform pooled analyses with larger sample size by summarizing previous case–control or cohort studies in order to better understand the association between the NKX2-3 variants and IBD risk.

NKX2-3, located on chromosome 10q24, is predominantly expressed in mesoderm of midgut and hindgut during embryonic mouse development^12^. Postnatally, Nkx2-3 expression continues in gut mesenchyme and in spleen. In addition, mice lacking Nkx2-3 exhibit severe defects in gut development; primarily in the epithelium of the small intestine^44^. The perturbations of the gut tissue architecture lead to early postnatal death presumably due to digestive malfunctions. Moreover, analysis of Nkx2-3-deficient mice has revealed a critical role for NKX2-3 in spleen development and in establishing the correct environment for normal B cell development and T cell dependent immune response^45^,^46^. Recently, associations between the two common polymorphisms (rs10883365 and rs11190140) in NKX2-3 gene and susceptibility of CD or UC have been reported in several studies.

To the best of our knowledge, the present study involving 37039 subjects represents the first comprehensive meta-analysis investigating the association between NKX2-3 polymorphisms (rs10883365 and rs11190140) and IBD susceptibility. For the analysis of rs10883365 polymorphism, a significantly increased CD risk was observed in all genetic models. In the ethnicity-stratified analyses, significant association was found both in Asian and European populations. Similar results were found between the rs11190140 variant and risk of CD where a significant association was found European population (no Asian population reported). No between-study heterogeneity was observed in most genetic models. Sensitivity analysis indicated that when excluding studies departed from HWE, the pooled OR still did not change, demonstrating the results of this meta-analysis were stable. Since these two SNPs are close to each other, we use 1000 Genomes Pilot sequence data to identify whether these SNPs are in linkage disequilibrium (LD) (r^2^ > 0.8). The results indicated that rs10883365 and rs11190140 are in perfect LD (r^2^ = 1.0).

CD and UC, as two major subtypes of IBD, are believed to share overlapping but distinct clinical and pathological features, and have great differences in genetic backgrounds^11^. Some genes, such as NOD2 and ATG16L1, were associated with CD, but not with UC^47^,^48^. However, recent GWAS meta-analysis identified 163 IBD loci that meet genome-wide significance thresholds, 50 of these have an indistinguishable effect size in UC and CD, including IL23R (rs11209026), IL10 (rs3024505) and MST1 (rs3197999). In the present meta-analysis, significant association between rs10883365 and risk of UC was found in all genetic models. When stratified by ethnicity, similar correlation was observed both in Asians and Europeans. No between-study heterogeneity existed in major genetic models. Sensitivity analysis showed no single study qualitatively changed the pooled ORs. Moreover, excluding studies departed from HWE, the pooled OR still did not change, demonstrating the results of this meta-analysis were stable. These results indicated that rs10883365 polymorphism in NKX2-3 gene may be significantly associated with both CD and UC.

By combining the data of individual studies, we increased the statistical power to detect subtle associations, however,several limitations should be considered in our meta-analysis. Only studies published in the English and Chinese language were included in this meta-analysis; therefore, publication bias may have occurred. In addition, this meta-analysis was designed to analyze single polymorphism, a haplotype analysis may have been more powerful for finding significant associations with UC and CD. Finally, gene–environment interactions were not analyzed because of insufficient data.

Despite these limitations, our results still yield statistical results. Taken together, we expand previously individual studies on IBD by suggesting that NKX2-3 gene rs10883365 polymorphisms might contribute to the occurrence of both CD and UC, and suggested that persons with a T allele of rs1190140 variant might have a significantly increased risk of CD. Further studies or large case-control studies, especially studies emphasizing genotype–phenotype interaction should be performed to clarify possible roles of NKX2-3in IBD. Moreover, studies involved in NKX2-3 polymorphisms in different populations with larger sample size might need to be performed.

## Methods

### Search strategy

We searched PubMed and Embase to identify genetic association studies of the rs10883365 or rs11190140 polymorphism and IBD risks. Electronic searches were performed by using the following search terms: ‘Inflammatory Bowel Disease' or ‘IBD', ‘Crohn's disease' or ‘CD', ‘ulcerative colitis' or ‘UC', ‘NKX2-3', ‘rs10883365', or ‘rs11190140', (the last search update was 1 September 2013). In addition, the reference lists of reviews and retrieved articles were checked by hand-search for additional potential studies. A study reported results from more than one population was considered as separate studies.

### Inclusion and exclusion criteria

Studies were considered eligible if they had to meet the following criteria: (1) association between NKX2-3 polymorphisms and risk of IBD (CD or UC), (2) case-control or cohort studies. Studies were excluded for the following reasons: (1) articles only with an abstract and review articles (2) no control population, (3) studies considered overlapped with other studies.

### Data extraction

Two authors extracted the following data independently from each of the eligible articles: first author, publication year, ethnicity of the participants involved (categorized as Europeans or Asians), number of cases and controls, information (age, mean age at diagnosis and sex) of cases and controls, and number of genotypes or allele frequency in cases and controls. Study authors were contacted for detailed data when there was insufficient information to determine the relationship between genetic polymorphism and IBD risk. Disagreements were resolved by discussion between the two authors.

### Statistical analysis

Pooled odds ratios (ORs) and 95% confidence intervals (CIs) used to assess the strength of the association between IBD risk (CD or UC) and NKX2-3 polymorphisms (rs10883365 or rs11190140). The significance of the pooled OR was determined by the Z-test; a P-value of <0.05 was considered significant. The Hardy-Weinberg equilibrium (HWE) in the control group was assessed, and a P < 0.05 was considered as significant disequilibrium. For rs10883365 polymorphism, the pooled ORs were estimated for G versus A, GG versus AA, GA versus AA, dominant model (GG + GA versus AA), and recessive model (GG versus GA + AA). Because of only three studies^19^,^49^,^50^ available for the association between rs11190140 variant and UC risk, we have performed meta-analysis of correlation between rs11190140 polymorphism and CD risk. Subgroup analysis was performed according to ethnicity.

Between-study heterogeneity was evaluated by using the Chi-square based Q test and I2 test^51^. Heterogeneity was considered significant for P < 0.10, and a random-effects model was used, otherwise, fixed-effects model was used. In addition, if heterogeneity was detected, Galbraith plots were used to visualize the impact of individual studies on the overall homogeneity, which spot the outliers as the possible major sources of heterogeneity^52^,^53^. Moreover, a meta-regression was used to delineate the major sources of between-study heterogeneity^54^.

Sensitivity analysis was carried out to evaluate the stability of the results after sequential removal of each study or by excluding those studies deviated from HWE. In addition, cumulative meta-analyses were carried out for each polymorphism through assortment of studies with publication time. Graphical evaluation of funnel plots and Egger's linear regression test were performed to assess publication bias^55^. If significant publication bias was detected, ORs and 95% CI would be adjusted by trim and fill methods^56^. All statistical analyses were performed by STATA software, version 12 (StataCorp LP, College Station, Texas).

## Supplementary Material

Supplementary Information supplementary figures

## Figures and Tables

**Table 1:** Main Characteristics of Studies Involved in NKX2-3 polymorphism and Crohn's disease or ulcerative colitis Risk

		Cases					Controls			
Author, Year of publishcation	Ethnicity	NKX2-3 variant	Phenotype Studied	Number	Males (%)	Age or Age at diagnosis	Number	Males (%)	Age	Matching
Tanaka, 2009	Asians	rs10883365	CD and UC	CD: 174	CD: 65.5	CD: 16–61	394	48.0	19–76	nr
			separately	UC: 296	UC: 48.0	UC: 15–79				
Meggyesi, 2010	Europeans	rs10883365	CD and UC separately	CD: 810	CD: 53.6	CD: 37.1 ± 12.6 and 26.5 ± 10.6 at diagnosis	469	53.5	40.5 ± 11.5	Age and sex
Meggyesi, 2010	Europeans	rs10883365	CD and UC separately	UC: 428	UC: 47.2	UC: 43.7 ± 15.0 and 31.3 ± 13.4 at diagnosis				
Fisher, 2008	Europeans	rs10883365	UC	UC: 1841	nr	nr	1470	nr	nr	nr
Franke, 2008	Europeans	rs10883365 rs11190140	CD and UC separately	CD: 1850UC: 1103	CD: 32.0	CD: mean 38 and 21at diagnosis	1817	nr	nr	Age and sex
Parkes, 2007	Europeans	rs10883365	CD	CD: 1182	CD: 40.3	CD: mean 43.9 and 25.5 at diagnosis	2024	nr	nr	nr
Parkes, 2007	Europeans	rs10883365	CD	CD: 1748	CD: 39.2	CD: mean 45.7 and 26.1 at diagnosis	5740	nr	nr	nr
Yu, 2009	Europeans	rs10883365	CD	CD: 75	nr	nr	255	nr	nr	nr
Yamazaki, 2009	Asians	rs10883365	CD	CD: 484	CD: 72.8	CD: 22.4 (7–55) at diagnosis	470	50.2	38.7(21–77)	
Pang, 2010	Asians	rs10883365	CD	CD: 66	CD: 48.5	CD: 36.26 ± 11.82	66	50.0	35.42 ± 13.14	Age and sex
Arai, 2011	Asians	rs10883365	CD and UC separately	CD: 344UC: 253	nrnr	nrnr	243	nr	nr	nr
Weersma, 2009	Europeans	rs10883365	CD and UC separately	CD: 1656UC: 1075	nr	nr	1086	nr	nr	nr
van der Heide, 2010	Europeans	rs10883365	CD	CD: 310	34.5	CD:26.6 (7.5–73.9) at diagnosis	976	nr	nr	nr
Latiano, 2011	Europeans	rs11190140	CD	CD: 1070	CD: 56%	nr	783	nr	nr	nr
Laukens, 2010	Europeans	rs11190140	CD	CD: 1051	nr	nr	676	nr	nr	nr
Peter, 2011	Europeans	rs11190140	CD	CD: 369	nr	nr	503	nr	nr	nr
Waterman, 2011	Europeans	rs11190140	CD	CD: 1144	CD: 53%	CD: 16(2–62) at diagnosis	1057	36%	nr	nr

NKX2-3: NK2 transcription factor related and locus 3; CD: Crohn's disease, UC: ulcerative colitis, nr: not report.

**Table 2:** Pooled analysis for the associations between the polymorphism of NKX2-3 and the risk of Crohn's disease

Variant	Comparison	Variables	No. of	Sample Size		Test of association		Model	Test of heterogeneity	
			studies	Case	Control	OR (95% CI)	P-value		I^2^(%)	P-value
rs10883365	G vs A	Overall	12	8699	13540	1.226 (1.177–1.277)	<0.001	F	0.0	0.968
		All in HWE	9	4883	9661	1.215 (1.154–1.280)	<0.001	F	0.0	0.913
		European	8	7631	12367	1.226 (1.174–1.280)	<0.001	F	0.0	0.944
		Asian	4	1068	1173	1.223 (1.082–1.382)	0.001	F	0.0	0.613
	GG vs AA	overall	10	6733	11478	1.481 (1.351–1.623)	<0.001	F	0.0	0.936
		All in HWE	9	4883	9661	1.476 (1.328–1.639)	<0.001	F	0.0	0.893
		European	6	5665	10305	1.481 (1.342–1.635)	<0.001	F	0.0	0.905
		Asian	4	1068	1173	1.477 (1.148–1.901)	0.002	F	0.0	0.566
	GA vs AA	Overall	10	6733	11478	1.141 (1.055–1.234)	0.001	F	0.0	0.836
		All in HWE	9	4883	9661	1.159 (1.059–1.268)	0.001	F	0.0	0.807
		European	6	5665	10305	1.116 (1.024–1.215)	0.012	F	0.0	0.796
		Asian	4	1068	1173	1.280 (1.055–1.553)	0.012	F	0.0	0.807
	GG + GA vs AA	Overall	10	6733	11478	1.241 (1.153–1.336)	<0.001	F	0.0	0.887
		All in HWE	9	4883	9661	1.254 (1.152–1.365)	<0.001	F	0.0	0.846
		European	6	5665	10305	1.225 (1.130–1.328)	<0.001	F	0.0	0.814
		Asian	4	1068	1173	1.328 (1.106–1.595)	0.002	F	0.0	0.693
	GG vs GA + GA	Overall	10	6733	11478	1.362 (1.263–1.468)	<0.001	F	0.0	0.960
		All in HWE	9	4883	9661	1.345 (1.235–1.465)	<0.001	F	0.0	0.948
		European	6	5665	10305	1.373 (1.268–1.486)	<0.001	F	0.0	0.927
		Asian	4	1068	1173	1.297 (1.024–1.598)	0.030	F	0.0	0.710
rs11190140	T vs C	Over(Europeans)	5	5484	4836	1.201 (1.136–1.269)	<0.001	F	0.0	0.773
		All in HWE	2	2121	1426	1.190 (1.080–1.311)	<0.001	F	0.0	0.544
	TT vs CC	Over(Europeans)	3	3971	3276	1.485 (1.297–1.700)	<0.001	F	0.0	0.631
		All in HWE	2	2121	1426	1.412 (1.162–1.716)	0.001	F	0.0	0.516
	TC vs CC	Over(Europeans)	3	3971	3276	1.155 (1.029–1.298)	0.015	F	0.0	0.478
		All in HWE	2	2121	1426	1.227 (1.033–1.458)	0.020	F	0.0	0.430
	TT + TC vs CC	Over(Europeans)	3	3971	3276	1.253 (1.122–1.398)	<0.001	F	0.0	0.867
		All in HWE	2	2121	1426	1.289 (1.095–1.516)	0.002	F	0.0	0.785
	TT vs TC + CC	Over(Europeans)	3	3971	3276	1.344 (1.204–1.501)	<0.001	F	50.1	0.135
		All in HWE	2	2121	1426	1.239 (1.061–1.446)	0.007	F	45.4	0.176

NKX2-3: NK2 transcription factor related and locus 3; R: Random-effects model, F: fixed-effects model, HWE: Hardy-Weinberg equilibrium; OR: odds ratio; CI: confidence interval.

**Table 3:** Pooled analysis for the associations between the polymorphism of NKX2-3 and the risk of ulcerative colitis

Variant	Comparison	Variables	No. of	Sample Size		Test of association		Model	Test of heterogeneity	
			studies	Case	Control	OR (95% CI)	P-value		I^2^(%)	P-value
rs10883365	G vs A	Overall	7	4996	5479	1.274 (1.175–1.382)	<0.001	R	44.5	0.094
		All in HWE	5	2818	2576	1.268 (1.174–1.369)	<0.001	F	36.5	0.178
		Europeans	5	4447	4842	1.225 (1.156–1.298)	<0.001	F	41.1	0.147
		Asians	2	549	637	1.452 (1.232–1.712)	<0.001	F	0.0	0.176
	GG vs AA	overall	6	3921	4393	1.672 (1.474–1.896)	<0.001	F	20.1	0.282
		All in HWE	5	2818	2576	1.619 (1.387–1.889)	<0.001	F	30.5	0.218
		Europeans	4	3372	3756	1.609 (1.404–1.844)	<0.001	F	26.0	0.255
		Asians	2	549	637	2.078 (1.500–2.878)	<0.001	F	0.0	0.654
	GA vs AA	Overall	6	3921	4393	1.207 (1.084–1.343)	0.001	F	0.0	0.901
		All in HWE	5	2818	2576	1.242 (1.090–1.416)	0.001	F	0.0	0.905
		Europeans	4	3372	3756	1.196 (1.063–1.345)	0.003	F	0.0	0.814
		Asians	2	549	637	1.260 (0.971–1.634)	0.082	F	0.0	0.466
	GG + GA vs AA	Overall	6	3921	4393	1.342 (1.213–1.485)	<0.001	F	0.0	0.801
		All in HWE	5	2818	2576	1.356 (1.199–1.533)	<0.001	F	0.0	0.688
		Europeans	4	3372	3756	1.317 (1.179–1.472)	<0.001	F	0.0	0.767
		Asians	2	549	637	1.467 (1.151–1.869)	0.002	F	0.0	0.454
	GG vs GA + GA	Overall	6	3921	4393	1.470 (1.325–1.630)	<0.001	F	43.9	0.112
		All in HWE	5	2818	2576	1.391 (1.223–1.581)	<0.001	F	41.2	0.146
		Europeans	4	3372	3756	1.455 (1.202–1.761)	<0.001	R	53.9	0.089
		Asians	2	549	637	1.534 (1.160–2.028)	<0.001	F	0.0	0.894

NKX2-3: NK2 transcription factor related and locus 3; R: Random-effects model, F: fixed-effects model, HWE: Hardy-Weinberg equilibrium; OR: odds ratio; CI: confidence interval.

## References

[b1] Vermeire S., Van Assche G. & Rutgeerts P. Classification of inflammatory bowel disease: the old and the new. Curr Opin Gastroenterol 28, 321–26 (2012). 22647554 10.1097/MOG.0b013e328354be1e

[b2] Abraham C. & Cho J. H. Inflammatory bowel disease. N Engl J Med 361, 2066–2078 (2009). 19923578 10.1056/NEJMra0804647 PMC3491806

[b3] McGovern D. P. *et al.* Genome-wide association identifies multiple ulcerative colitis susceptibility loci. Nat Genet 42, 332–337 (2010). 20228799 10.1038/ng.549 PMC3087600

[b4] Tysk C., Lindberg E., Jarnerot G. & Floderus-Myrhed B. Ulcerative colitis and Crohn's disease in an unselected population of monozygotic and dizygotic twins. A study of heritability and the influence of smoking. Gut 29, 990–996 (1988). 3396969 10.1136/gut.29.7.990 PMC1433769

[b5] Colombel J. F. *et al.* Clinical characteristics of Crohn's disease in 72 families. Gastroenterology 111, 604–607 (1996). 8780563 10.1053/gast.1996.v111.pm8780563

[b6] Glas J. *et al.* IRGM variants and susceptibility to inflammatory bowel disease in the German population. PLoS One 8, e54338 (2013). 23365659 10.1371/journal.pone.0054338 PMC3554777

[b7] Molnar T. *et al.* NOD1 gene E266K polymorphism is associated with disease susceptibility but not with disease phenotype or NOD2/CARD15 in Hungarian patients with Crohn's disease. Dig Liver Dis 39, 1064–1070 (2007). 17964870 10.1016/j.dld.2007.09.003

[b8] Lakatos P. L. *et al.* ATG16L1 and IL23 receptor (IL23R) genes are associated with disease susceptibility in Hungarian CD patients. Dig Liver Dis 40, 867–873 (2008). 18499543 10.1016/j.dld.2008.03.022

[b9] Kabi A., Nickerson K. P., Homer C. R. & McDonald C. Digesting the genetics of inflammatory bowel disease: insights from studies of autophagy risk genes. Inflamm Bowel Dis 18, 782–792 (2012). 21936032 10.1002/ibd.21868 PMC3245781

[b10] Scharl M. *et al.* Crohn's disease-associated polymorphism within the PTPN2 gene affects muramyl-dipeptide-induced cytokine secretion and autophagy. Inflamm Bowel Dis 18, 900–912 (2012). 22021207 10.1002/ibd.21913

[b11] Waterman M. *et al.* Distinct and overlapping genetic loci in Crohn's disease and ulcerative colitis: correlations with pathogenesis. Inflamm Bowel Dis 17, 1936–1942 (2011). 21830272 10.1002/ibd.21579 PMC3164287

[b12] Pabst O., Schneider A., Brand T. & Arnold H. H. The mouse Nkx2-3 homeodomain gene is expressed in gut mesenchyme during pre- and postnatal mouse development. Dev Dyn 209, 29–35 (1997). 9142493 10.1002/(SICI)1097-0177(199705)209:1<29::AID-AJA3>3.0.CO;2-Z

[b13] Fu Y., Yan W., Mohun T. J. & Evans S. M. Vertebrate tinman homologues XNkx2-3 and XNkx2-5 are required for heart formation in a functionally redundant manner. Development 125, 4439–4449 (1998). 9778503 10.1242/dev.125.22.4439

[b14] Genome-wide association study of 14,000 cases of seven common diseases and 3,000 shared controls. Nature 447, 661–678 (2007). 17554300 10.1038/nature05911 PMC2719288

[b15] Meggyesi N. *et al.* NKX2-3 and IRGM variants are associated with disease susceptibility to IBD in Eastern European patients. World J Gastroenterol 16, 5233–5240 (2010). 21049557 10.3748/wjg.v16.i41.5233 PMC2975094

[b16] Parkes M. *et al.* Sequence variants in the autophagy gene IRGM and multiple other replicating loci contribute to Crohn's disease susceptibility. Nat Genet 39, 830–832 (2007). 17554261 10.1038/ng2061 PMC2628541

[b17] Arai T. *et al.* Increased expression of NKX2.3 mRNA transcribed from the risk haplotype for ulcerative colitis in the involved colonic mucosa. Hum Immunol 72, 587–591 (2011). 21514341 10.1016/j.humimm.2011.03.023

[b18] Fisher S. A. *et al.* Genetic determinants of ulcerative colitis include the ECM1 locus and five loci implicated in Crohn's disease. Nat Genet 40, 710–712 (2008). 18438406 10.1038/ng.145 PMC2719289

[b19] Franke A. *et al.* Replication of signals from recent studies of Crohn's disease identifies previously unknown disease loci for ulcerative colitis. Nat Genet 40, 713–715 (2008). 18438405 10.1038/ng.148

[b20] Tanaka M. *et al.* Genetic variants in surfactant, pulmonary-associated protein D (SFTPD) and Japanese susceptibility to ulcerative colitis. Inflamm Bowel Dis 15, 918–925 (2009). 19340882 10.1002/ibd.20936

[b21] Pang Z., Cao K. & Wei W. X. Correlation of rs10883365 Polymorphism in Upstream Region of NKX2-3 Gene with Crohn' Disease in Chinese Han Population. Chin J Gastroenterol 15, 532–535 (2010). (Chinese).

[b22] Latiano A. *et al.* Investigation of multiple susceptibility loci for inflammatory bowel disease in an Italian cohort of patients. PLoS One 6, e22688 (2011). 21818367 10.1371/journal.pone.0022688 PMC3144927

[b23] Yu W. *et al.* Association of a Nkx2-3 polymorphism with Crohn's disease and expression of Nkx2-3 is up-regulated in B cell lines and intestinal tissues with Crohn's disease. J Crohns Colitis 3, 189–195 (2009). 21172269 10.1016/j.crohns.2009.04.003

[b24] Weersma R. K. *et al.* Confirmation of multiple Crohn's disease susceptibility loci in a large Dutch-Belgian cohort. Am J Gastroenterol 104, 630–638 (2009). 19174780 10.1038/ajg.2008.112

[b25] Yamazaki K. *et al.* Positive association of genetic variants in the upstream region of NKX2-3 with Crohn's disease in Japanese patients. Gut 58, 228–232 (2009). 18936107 10.1136/gut.2007.140764

[b26] Laukens D. *et al.* Evidence for significant overlap between common risk variants for Crohn's disease and ankylosing spondylitis. PLoS One 5, e13795 (2010). 21072187 10.1371/journal.pone.0013795 PMC2970560

[b27] van der Heide F. *et al.* Differences in genetic background between active smokers, passive smokers, and non-smokers with Crohn's disease. Am J Gastroenterol 105, 1165–1172 (2010). 19953089 10.1038/ajg.2009.659

[b28] Peter I. *et al.* Evaluation of 22 genetic variants with Crohn's disease risk in the Ashkenazi Jewish population: a case-control study. BMC Med Genet 12, 63 (2011). 21548950 10.1186/1471-2350-12-63 PMC3212904

[b29] Duerr R. H. *et al.* A genome-wide association study identifies IL23R as an inflammatory bowel disease gene. Science 314, 1461–1463 (2006). 17068223 10.1126/science.1135245 PMC4410764

[b30] Imielinski M. *et al.* Common variants at five new loci associated with early-onset inflammatory bowel disease. Nat Genet 41, 1335–1340 (2009). 19915574 10.1038/ng.489 PMC3267927

[b31] Franke A. *et al.* Genome-wide meta-analysis increases to 71 the number of confirmed Crohn's disease susceptibility loci. Nat Genet 42, 1118–1125 (2010). 21102463 10.1038/ng.717 PMC3299551

[b32] Anderson C. A. *et al.* Meta-analysis identifies 29 additional ulcerative colitis risk loci, increasing the number of confirmed associations to 47. Nat Genet 43, 246–252 (2011). 21297633 10.1038/ng.764 PMC3084597

[b33] Jostins L. *et al.* Host-microbe interactions have shaped the genetic architecture of inflammatory bowel disease. Nature 491, 119–124 (2012). 23128233 10.1038/nature11582 PMC3491803

[b34] Cheng J. F., Ning Y. J., Zhang W., Lu Z. H. & Lin L. T300A polymorphism of ATG16L1 and susceptibility to inflammatory bowel diseases: a meta-analysis. World J Gastroenterol 16, 1258–1266 (2010). 20222171 10.3748/wjg.v16.i10.1258 PMC2839180

[b35] Fan W. *et al.* Relationship between the polymorphism of tumor necrosis factor-alpha-308 G > A and susceptibility to inflammatory bowel diseases and colorectal cancer: a meta-analysis. Eur J Hum Genet 19, 432–437 (2011). 21248737 10.1038/ejhg.2010.159 PMC3060311

[b36] Shen Y. *et al.* The −173 G/C Polymorphism of the MIF Gene and Inflammatory Bowel Disease Risk: A Meta-Analysis. Int J Mol Sci 14, 11392–11401 (2013). 23759989 10.3390/ijms140611392 PMC3709738

[b37] Xuan C. *et al.* Association between OCTN1/2 gene polymorphisms (1672C-T, 207G-C) and susceptibility of Crohn's disease: a meta-analysis. Int J Colorectal Dis 27, 11–19 (2012). 21706137 10.1007/s00384-011-1265-x

[b38] Wang Z., Hu J., Fan R., Zhou J. & Zhong J. Association between CD14 gene C-260T polymorphism and inflammatory bowel disease: a meta-analysis. PLoS One 7, e45144 (2012). 23049772 10.1371/journal.pone.0045144 PMC3458839

[b39] Zintzaras E. Is there evidence to claim or deny association between variants of the multidrug resistance gene (MDR1 or ABCB1) and inflammatory bowel disease? Inflamm Bowel Dis 18, 562–572 (2012). 21887726 10.1002/ibd.21728

[b40] Lu W. G. *et al.* Association of NOD1 (CARD4) insertion/deletion polymorphism with susceptibility to IBD: a meta-analysis. World J Gastroenterol 16, 4348–4356 (2010). 20818820 10.3748/wjg.v16.i34.4348 PMC2937117

[b41] Zhang J. X. *et al.* Associations between PTPN2 polymorphisms and susceptibility to ulcerative colitis and Crohn's disease: a meta-analysis. Inflamm Res 63, 71–79 (2014). 24127071 10.1007/s00011-013-0673-5

[b42] Zou L. *et al.* The association between three promoter polymorphisms of IL-10 and inflammatory bowel diseases (IBD): A meta-analysis. Autoimmunity 47, 27–39 (2014). 24128120 10.3109/08916934.2013.843672

[b43] Pan H. F., Leng R. X. & Ye D. Q. Lack of association of interleukin-18 gene promoter −607 A/C polymorphism with susceptibility to autoimmune diseases: a meta-analysis. Lupus 20, 945–951 (2011). 21636628 10.1177/0961203311400114

[b44] Pabst O., Zweigerdt R. & Arnold H. H. Targeted disruption of the homeobox transcription factor Nkx2-3 in mice results in postnatal lethality and abnormal development of small intestine and spleen. Development 126, 2215–2225 (1999). 10207146 10.1242/dev.126.10.2215

[b45] Pabst O., Forster R., Lipp M., Engel H. & Arnold H. H. NKX2.3 is required for MAdCAM-1 expression and homing of lymphocytes in spleen and mucosa-associated lymphoid tissue. EMBO J 19, 2015–2023 (2000). 10790368 10.1093/emboj/19.9.2015 PMC305695

[b46] Tarlinton D., Light A., Metcalf D., Harvey R. P. & Robb L. Architectural defects in the spleens of Nkx2-3-deficient mice are intrinsic and associated with defects in both B cell maturation and T cell-dependent immune responses. J Immunol 170, 4002–4010 (2003). 12682228 10.4049/jimmunol.170.8.4002

[b47] Yazdanyar S., Weischer M. & Nordestgaard B. G. Genotyping for NOD2 genetic variants and crohn disease: a metaanalysis. Clin Chem 55, 1950–1957 (2009). 19713276 10.1373/clinchem.2009.127126

[b48] Marquez A. *et al.* Role of ATG16L1 Thr300Ala polymorphism in inflammatory bowel disease: a Study in the Spanish population and a meta-analysis. Inflamm Bowel Dis 15, 1697–1704 (2009). 19575361 10.1002/ibd.21001

[b49] John G. *et al.* NKX2-3 variant rs11190140 is associated with IBD and alters binding of NFAT. Mol Genet Metab 104, 174–179 (2011). 21803625 10.1016/j.ymgme.2011.06.023

[b50] Skieceviciene J. *et al.* Replication Study of Ulcerative Colitis Risk Loci in a Lithuanian-Latvian Case-Control Sample. Inflamm Bowel Dis 19, 2349–2355 (2013). 23974994 10.1097/MIB.0b013e3182a3eaeb

[b51] Higgins J. P. & Thompson S. G. Quantifying heterogeneity in a meta-analysis. Stat Med 21, 1539–1558 (2002). 12111919 10.1002/sim.1186

[b52] Galbraith R. F. A note on graphical presentation of estimated odds ratios from several clinical trials. Stat Med 7, 889–894 (1988). 3413368 10.1002/sim.4780070807

[b53] Huy N. T. *et al.* Cerebrospinal fluid lactate concentration to distinguish bacterial from aseptic meningitis: a systemic review and meta-analysis. Crit Care 14, R240 (2010). 21194480 10.1186/cc9395 PMC3220013

[b54] Kriston L., Harms A. & Berner M. M. A meta-regression analysis of treatment effect modifiers in trials with flexible-dose oral sildenafil for erectile dysfunction in broad-spectrum populations. Int J Impot Res 18, 559–565 (2006). 16688210 10.1038/sj.ijir.3901479

[b55] Tang J. L. & Liu J. L. Misleading funnel plot for detection of bias in meta-analysis. J Clin Epidemiol 53, 477–484 (2000). 10812319 10.1016/s0895-4356(99)00204-8

[b56] Fan W. *et al.* Relationship between the polymorphism of tumor necrosis factor-alpha-308 G > A and susceptibility to inflammatory bowel diseases and colorectal cancer: a meta-analysis. Eur J Hum Genet 19, 432–437 (2011). 21248737 10.1038/ejhg.2010.159 PMC3060311

