# Supplementary material for: Contribution of NKX2-3 Polymorphisms to Inflammatory Bowel Diseases: A Meta-Analysis of 35358 subjects
Source: Sci Rep. 2014 Jan 29;4:3924. doi: 10.1038/srep03924 (PMC5379238; doi:10.1038/srep03924)
Supplement: Supplementary Information — supplementary figures [file srep03924-s1.doc]

**Contribution of NKX2-3 Polymorphisms to Inflammatory Bowel Diseases: A Meta-Analysis of 35358 subjects**

Xiao-Cheng Lu, Linjun Tang, Kai Li, Jin-Yu Zheng, Penglai Zhao, Yi Tao, Li-Xin Li*

Department of Neurosurgery, First Affiliated Hospital of Nanjing Medical University, 300 Guangzhou Road, Nanjing, Jiangsu, 210029, China

*Corresponding author

**Supplementary Figure S1**


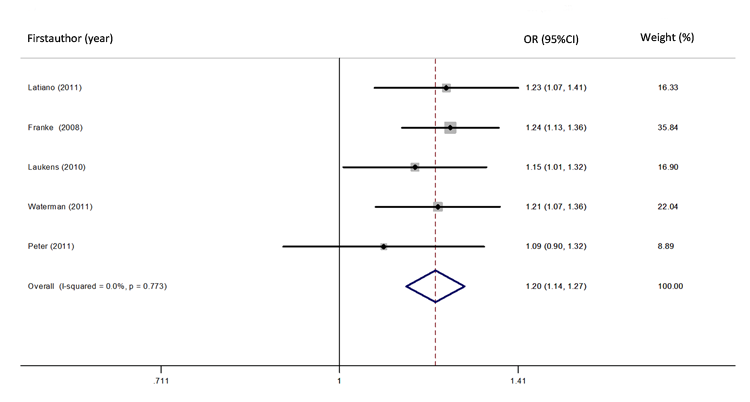


**Supplementary Figure S1**. OR estimates with the corresponding 95% CI for the association between rs11190140 polymorphism in NKX2-3 gene and CD risk. (T vs C) The sizes of the squares reflect the weighting of included studies. OR: odds ratio; CI: confidence interval.

**Supplementary Figure S2**


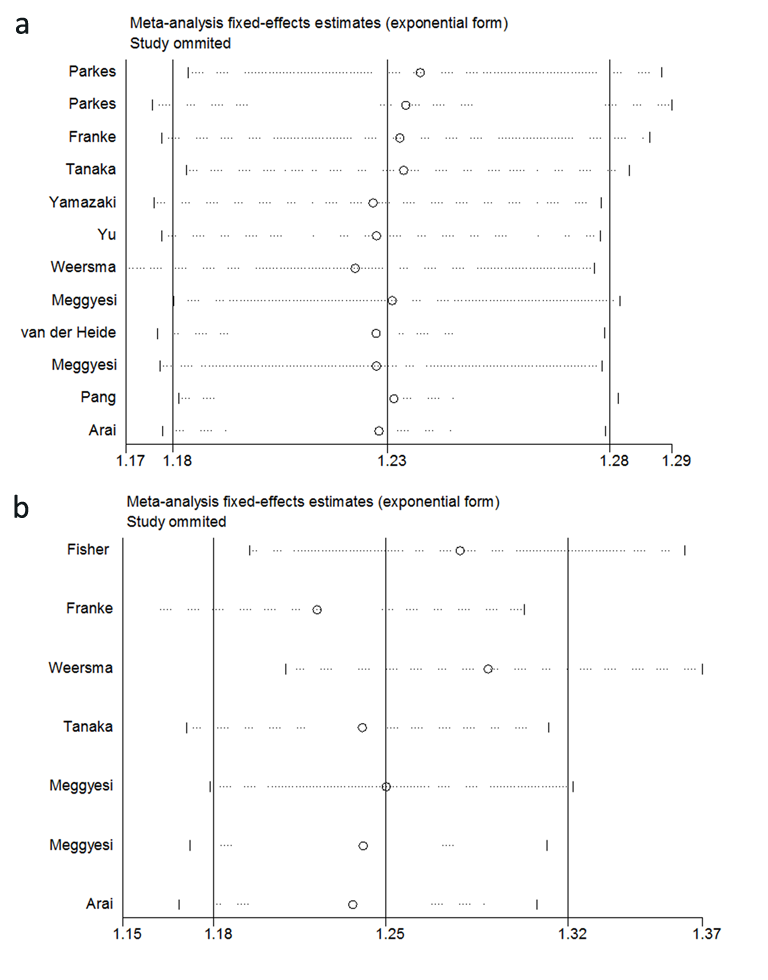


**Supplementary Figure S2.** Sensitivity analysis on the association between rs10883365 polymorphism and CD or UC risk. (A): sensitivity analysis of rs10883365 polymorphism and CD risk (G vs. A); (B): sensitivity analysis of rs10883365 polymorphism and UC risk (G vs. A); Results were computed by omitting each study (left column) in turn. CD: Crohn’s disease; UC: ulcerative colitis; Bars: 95% confidence interval.

**Supplementary Figure S3**


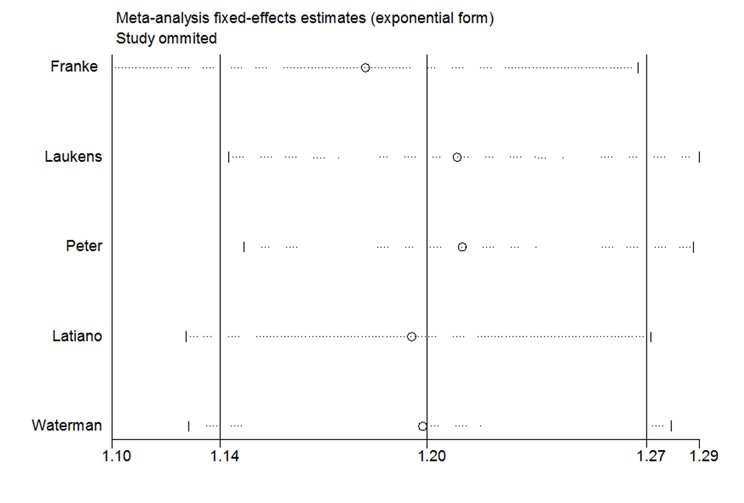


**Supplementary Figure S3.** Sensitivity analysis on the association between rs11190140 polymorphism and CD risk. (T vs C) Results were computed by omitting each study (left column) in turn. CD: Crohn’s disease; UC: ulcerative colitis; Bars: 95% confidence interval.

**Supplementary Figure S4**


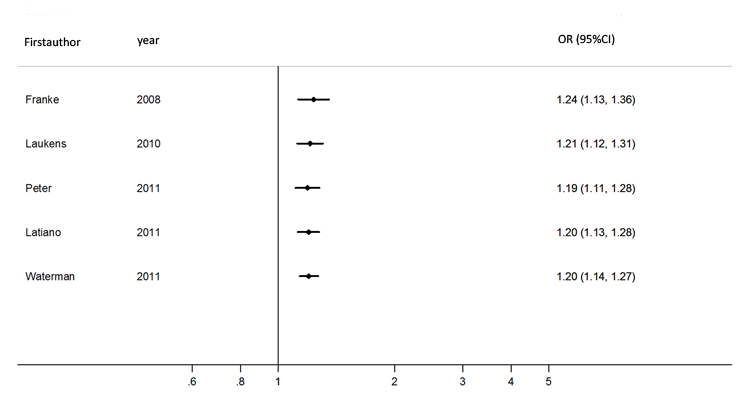


**Supplementary Figure S4.** Cumulative meta-analysis on the association between rs11190140 polymorphism and CD risk. (T vs C) Pooled OR estimates with the 95% CI as information accumulates at the end of each year (left column). CD: Crohn’s disease; UC: ulcerative colitis; OR: odds ratio; CI: confidence interval.

**Supplementary Figure S5**


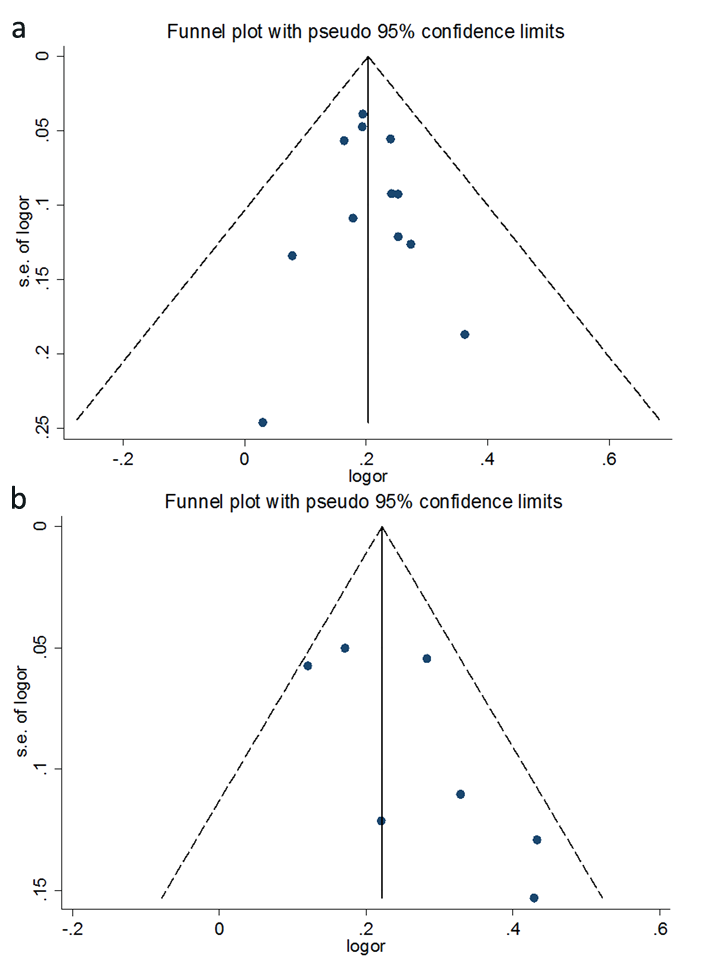


**Supplementary Figure S5.** Publication bias in studies of the association between rs10883365 polymorphism and CD or UC risk assessed by Funnel plot. (A): rs10883365 variant and CD risk (GA vs. AA); B: rs10883365 variant and UC risk (GA vs. AA).No significant funnel asymmetry was observed which could indicate publication bias. The vertical line in the funnel plot indicates the summary estimate, while the sloping lines indicate the expected 95 % CI for a given standard error, assuming no heterogeneity between studies. logor Natural logarithm of the OR, s.e. of logor standard error of the logOR. OR: Odds Ratio; CI: confidence interval.

**Supplementary Figure S6**


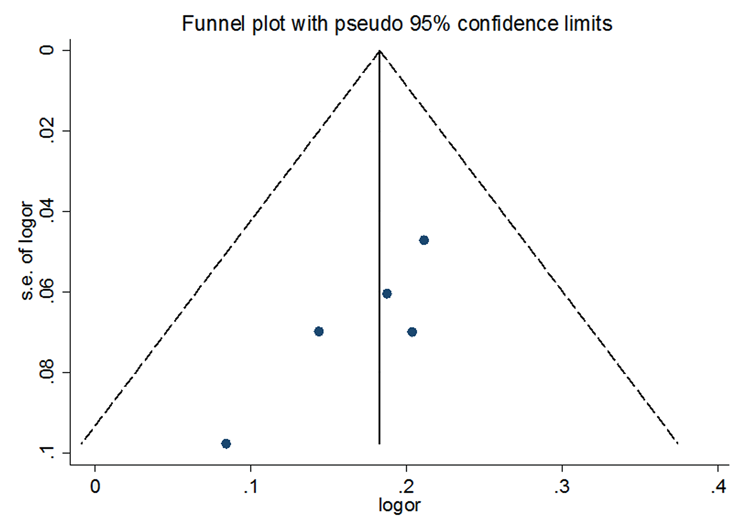


**Supplementary Figure S6.** Publication bias in studies of the association between rs11190140 polymorphism and CD risk assessed by Funnel plot. (T vs C) No significant funnel asymmetry was observed which could indicate publication bias. The vertical line in the funnel plot indicates the summary estimate, while the sloping lines indicate the expected 95 % CI for a given standard error, assuming no heterogeneity between studies. logor Natural logarithm of the OR, s.e. of logor standard error of the logOR. OR: Odds Ratio; CI: confidence interval
